# The association between employee lifestyles and the rates of mental health-related absenteeism and turnover in Japanese companies

**DOI:** 10.4178/epih.e2024068

**Published:** 2024-08-02

**Authors:** Atsuya Fujimoto, Hiroshi Kanegae, Kaori Kitaoka, Mizuki Ohashi, Kunio Okada, Koichi Node, Kenkichi Takase, Hiroshi Fukuda, Tomoyuki Miyazaki, Yuichiro Yano

**Affiliations:** 1 Center for Promotion of Research and Industry-Academic Collaboration, Department of Core Project Promotion, Yokohama City University, Yokohama, Japan; 2 Office of Research and Analysis, Genki Plaza Medical Center for Health Care, Tokyo, Japan; 3 Noncommunicable Disease (NCD) Epidemiology Research Center, Shiga University of Medical Science, Shiga, Japan; 4 Nonprofit Organization Kenkokeiei, Tokyo, Japan; 5 Department of Cardiovascular Medicine, Saga University, Saga, Japan; 6 Department of Psychology, Chuo University, Tokyo, Japan; 7 Department of General Medicine, Juntendo University Faculty of Medicine, Tokyo, Japan; 8 Department of Safety and Health Promotion, Juntendo University, Tokyo, Japan; 9 Department of Family Medicine and Community Health, Duke University, NC, USA

**Keywords:** Employee, Lifestyles, Mental health, Absenteeism

## Abstract

We assessed the association of employee lifestyles (e.g., smoking, exercise, drinking, and sleep habits) with mental health-related absenteeism and turnover rates utilizing data from the annual Health and Productivity Management survey by Japan’s Ministry of Economy, Trade and Industry. This analysis included data from 1,748 companies, encompassing 4,199,021 employees. The average proportions of mental health-related absenteeism and employee turnover rates were 1.1±1.0% and 5.0±5.0%, respectively. In multivariable regression models that incorporated all lifestyle factors and confounders, a 1 percentage point increase in the proportion of employees who slept well was associated with reductions in their turnover rate (mean, -0.020%; 95% confidence interval [CI], -0.038 to -0.002) and in mental health-related absenteeism (mean, -0.005%; 95% CI, -0.009 to 0.001). A similar increase in the proportion of employees engaging in regular physical activity corresponded with a 0.005% decrease in the prevalence of mental health-related absenteeism (95% CI, -0.010 to -0.001). A 1 percentage point increase in the proportion of employees who smoked was associated with a 0.013% reduction in mental health-related absenteeism (95% CI, -0.017 to -0.008). Nonetheless, the current study’s observational and cross-sectional design restricted the ability to establish causality between employee lifestyle factors and mental health issues.

## GRAPHICAL ABSTRACT

[Fig f1-epih-46-e2024068]

## Key Message

Utilizing data from the annual Health and Productivity Management survey conducted by Japan’s Ministry of Economy, Trade and Industry, we evaluated the relationship between employees lifestyles and mental health-related absenteeism and turnover rates.We found sleep, exercise, and smoking habits were associated with mental health-related absenteeism and turnover rates.Improving employee lifestyle may contribute to a reduction in mental health-related absenteeism and turnover rates.

## INTRODUCTION

The worldwide prevalence of mental health problems is high and rising [1], with 970 million people suffering from mental health problems according to the World Health Organization [2]. In Japan, the lifetime prevalence of mental health problems is over 20% [3]. Furthermore, a systematic review and meta-analysis reported that the pooled prevalence of mental health problems during the coronavirus disease 2019 pandemic was higher than prior to the outbreak [4,5]. Mental health disorders in the workplace, such as depression and anxiety, have increasingly been recognized as a problem in most countries. Mental health disorders result in significant costs, not only the immediate costs associated with medical treatment, healthcare visits, and hospitalization, but also the indirect financial burdens. These include lost wages and reduced work efficiency due to disabilities that can lead to both absenteeism (i.e., decreased number of days away from work) and presenteeism (i.e., diminished productivity at work) [6]. These costs exacerbate conditions of poverty [7], a vulnerability that in turn worsens mental health, feeding a vicious cycle of poverty and illness [8]. At the national and global levels, mental health disorders reduce the supply of labor and capital, resulting in lower economic output [9,10].

Previous studies have provided evidence that poor mental health among employees is linked to reduced productivity, as evidenced by increased rates of absenteeism and presenteeism [11-13]. Furthermore, poor mental health among employees is associated with higher turnover rates, which can be costly for employers [14,15]. A healthier lifestyle may be associated with fewer mental health issues, which could contribute to lower employee turnover rates [16]. However, these associations have been little studied in Japan [17]. Utilizing data from the annual Health and Productivity Management survey conducted by Japan’s Ministry of Economy, Trade and Industry (METI), we assessed the associations of employee lifestyle, including smoking, exercise, drinking, and sleep habits, with mental health-related absenteeism and turnover rates in Japanese companies.

## MATERIALS AND METHODS

The METI has defined the essential elements of an integrated health protection and health promotion model to create a healthier, high-performing workforce [18]. METI and the Tokyo Stock Exchange (TSE) are encouraging companies to further strengthen health and productivity management efforts. METI conducted an annual survey on Health and Productivity Management; the survey design has been described elsewhere [17]. In Japan, the Industrial Safety and Health Act mandates that employers must ensure that their employees undergo annual medical examinations conducted by a physician. During these health check-ups, employees are required to complete a standardized questionnaire concerning their lifestyle habits. The data collection is typically managed by the company’s human resources or health management departments, which report the proportions of various lifestyle habits. This centralized reporting method helps to provide an accurate reflection of the health status of the workforce.

In the current study, we analyzed data from a survey conducted in fiscal year 2020, from April 2019 to March 2020. We analyzed data from 1,748 companies that submitted information about employee lifestyles, mental health-related absenteeism, and turnover rates. We provide a summary of the survey questions used in our analysis here:

- Is your company listed on a stock exchange? (Options: Yes or No)

- Please select the type of industry in which your company operates (Options: Wholesale, Retail, Service, Other)

- Please describe the average age and length of service of fulltime employees (Include the absolute numbers)

- What is the percentage of employees in your company who have a habit of regular exercise for more than 30 minutes at least twice a week? (Include the percentage)

- What is the percentage of employees in your company who sleep well? (Include the percentage)

- What percentage of employees in your company have a body mass index between 18.5 kg/m2 and 25.0 kg/m2 ? (Include the percentage)

- What is the percentage of current smokers in your company? (Include the percentage)

- What is the percentage of current alcohol drinkers in your company? (Include the percentage)

- Please describe the number of full-time employees and parttime employees (Include the percentage)

- Please describe the number of full-time employees who left the organization during the fiscal year (Include the absolute numbers)

- Does your company assess the number of full-time employees who are absent or on leave from work for at least 1 month due to mental health issues? (Include the absolute numbers)

### Statistical analysis

The outcomes of this study included (1) the employee turnover rate, calculated as the percentage of full-time employees who left the organization during the fiscal year, divided by the total number of full-time employees at the start of the year, and (2) the proportion of mental health-related absenteeism among employees, calculated by dividing the number of full-time employees who were absent from work due to mental health issues, by the total number of full-time employees, expressed as a percentage.

The descriptive statistics were represented as totals and percentages. To assess the association of employee lifestyles with the rates of mental health-related absenteeism and turnover, we used linear regression models. Each model calculated regression coefficients with 95% confidence intervals (CIs) for lifestyle factors, such as maintaining a healthy weight, sleep quality, level of physical activity, and smoking and alcohol consumption. Furthermore, multivariable regression models, incorporating all lifestyle factors and confounders, were conducted. These confounders included the company’s listing status on the stock exchange, industry type, and the average tenure of full-time employees. Statistical significance was established at a p-value < 0.05, using two-tailed tests. All analyses were performed using SAS version 9.4 (SAS Institute Inc., Cary, NC, USA).

### Ethics statement

The study was approved by the Ethics Committee of Shiga University of Medical Science (RRB21-053-3) and was in accordance with the principles of the Declaration of Helsinki. In accordance with the Ethical Guidelines for Medical and Health Research Involving Human Subjects in Japan, we did not obtain individual informed consent because the data was anonymized (i.e., survey questions for the companies).

## RESULTS

The database included survey results from 1,748 companies (n=4,199,021 employees). The types of industries represented included construction (n=159,398, 3.8%), food service (n=113,954, 2.7%), chemical processing (n=175,758, 4.2%), electrical manufacturing (n=499,312, 11.9%), transportation equipment (n=446,637, 10.6%), shipping (n=207,596, 4.9%), telecommunication (n=373,832, 8.9%), wholesale (n=135,843, 3.2%), retail (n=258,209, 6.1%), financial services (n =646,990, 15.4%), professional services (n=239,349, 5.7%), and other (n=942,143, 22.4%). Among the 1,748 companies surveyed, the proportion of female employees was 26.8%, the mean length of service was 14.4± 4.6 years, and the rates of mental health-related absenteeism and employee turnover were 1.1± 1.0% and 5.0± 5.0%, respectively.

In Table 1, a 1 percentage point increase in the proportion of employees who smoked (mean, -0.030%; 95% CI, -0.053 to -0.006), slept well (mean, -0.024%; 95% CI, -0.044 to -0.004), and consumed alcohol (mean, -0.029%; 95% CI, -0.049 to -0.009) was associated with their turnover rate (unadjusted models). When all lifestyle variables were included in the same model (including confounders), the association between sleep and turnover rates remained significant (adjusted model).

In Table 2, a 1 percentage point increase in the proportion of employees who smoked (mean, -0.015%; 95% CI, -0.020 to -0.011), exercised regularly (mean, -0.008%; 95% CI, -0.012 to -0.003), slept well (mean, -0.006%; 95% CI, -0.010 to -0.002), and consumed alcohol (mean, -0.008%; 95% CI, -0.012 to -0.004) was associated with a reduction in mental health-related absenteeism (unadjusted models). When all lifestyle factors (including confounders) were included in the same model, the associations of smoking, regular exercise, and sleeping well with mental health-related absenteeism remained statistically significant (adjusted model).

## DISCUSSION

Using data from a survey on national health and productivity management, we found that a 1 percentage point increase in the proportion of employees who slept well was associated with a statistically significant reduction in the rates of employee turnover and mental health-related absenteeism. Specifically, the decrease in turnover rate was -0.020% (95% CI, -0.038 to -0.002), and the reduction in mental health-related absenteeism was -0.005% (95% CI, -0.009 to -0.001). In addition, a 1 percentage point increase in the proportion of employees who exercised regularly was associated with a reduction in mental health-related absenteeism by -0.005% (95% CI, -0.010 to -0.001). Considering that, among the 1,748 companies surveyed, the average proportion of mental healthrelated absenteeism was 1.1 ± 1.0%, and the average employee turnover rate was 5.0± 5.0%, the reductions in both turnover rate and mental health-related absenteeism in the current study appear to be significant from a practical standpoint.

Litwiller et al. [19] conducted a meta-analysis using data from 152 primary studies on sleep among workers in organizations, examining the associations of sleep quality and sleep quantity with work performance. It was found that both aspects of sleep were negatively correlated with workload and various health, attitudinal, and affective outcomes. Nagamatsu [20] conducted a literature review to examine the association of exercise with employee mental health. Despite the limitations of the reviewed studies, such as their cross-sectional design, small scale, and inconsistent measurement methods, the findings indicated that exercise may have a positive effect on employees’ affective outcomes. Recreational physical activities were also shown to help reduce chronic stress. Conversely, prolonged periods of sedentary behavior were associated with poorer mental health outcomes. These reviews highlight the potential benefits of adopting organizational policies and practices that promote healthy sleep habits and encourage physical activity. Such initiatives may enhance mental well-being in the workplace, demonstrating the importance of integrating wellness strategies into the corporate culture to improve employee health and productivity.

A one-percentage point increase in the proportion of employees who smoked was associated with a 0.013% reduction in mental health-related absenteeism (95% CI, -0.017 to -0.008). The association between smoking and mental health has been a subject of ongoing debate. The self-medication hypothesis, which posits that smoking may alleviate symptoms of mental health disorders, is rooted in psychodynamic theory [21]. Empirical support for this hypothesis includes findings from randomized human trials indicating that nicotine enhances short-term memory, attention, and other cognitive functions [22]. In addition, nicotine has been shown to induce the release of neurotransmitters that enhance feelings of reward and pleasure [23,24]. However, longitudinal studies indicate that smoking exacerbates mental health problems, while cessation is linked with mental health improvements [25]. This apparent contradiction may be explained by the misattribution hypothesis, suggesting that smokers confuse the relief from tobacco withdrawal symptoms with an alleviation of mental health symptoms. Supporting this, a comprehensive Cochrane systematic review by Taylor et al. [26-28], consistently found that smoking cessation correlated with mental health improvements, with effect sizes comparable to those of antidepressants. The limitations of the current study’s observational and cross-sectional design must be acknowledged, as they restrict the ability to establish causality between smoking and mental health issues.

The current study had several limitations. First, the annual Health and Productivity Management survey was conducted by METI, which grants awards to companies that excel in providing health protection and promoting health among employees, aiming to foster a healthier, high-performing workforce. Since the survey responses were based on aggregated data, we did not have access to individual employee data. Although the current study, with its ecological design, provides insights into trends at the group level, caution should be exercised when interpreting these findings at the individual level. Second, the lifestyle-related questionnaires collected from employees were derived from a standardized national format, which likely accounts for their simplistic structure. In addition, these questionnaires depended on subjective self-reports rather than objective data. For instance, perceptions of “sleeping well” might range from 6 hours of sleep per night for some to 8 hours for others, yet there was no objective, quantitative measure to assess sleep quality included in these surveys. Third, we did not include other unmeasured factors, such as the economic status of the company or subjective assessments of its quality, when examining the associations between employee lifestyles and rates of mental health-related absenteeism and turnover.

## Figures and Tables

**Figure f1-epih-46-e2024068:**
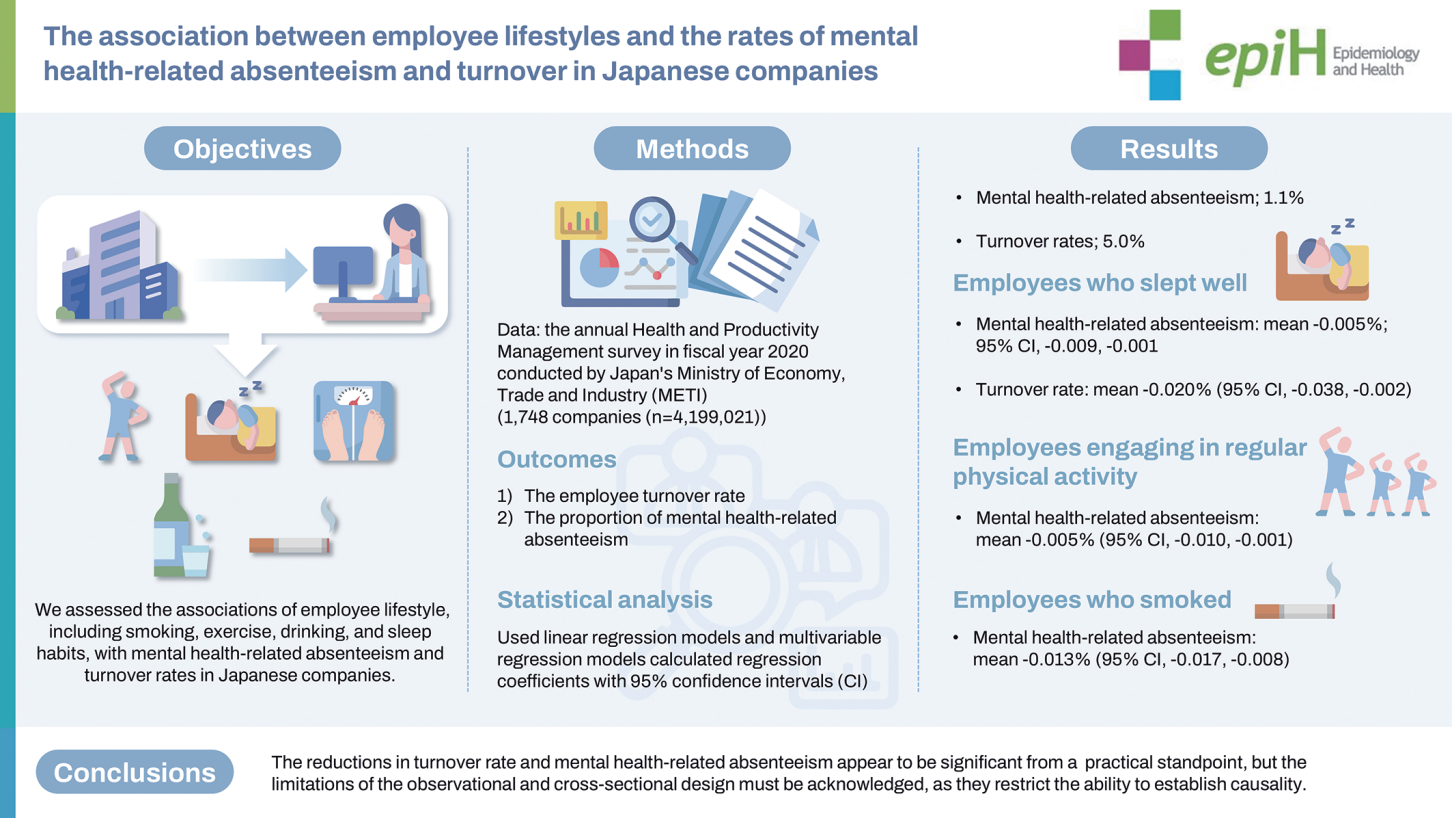


**Table 1.** Association of employee lifestyle factors with the turnover rate among 1,748 Japanese companies^1^

Variables (1%)	Unadjusted model	p-value	Adjusted model	p-value
Maintenance of appropriate weight	0.023 (-0.013, 0.060)	0.206	-0.014 (-0.047, 0.019)	0.396
Smoking	-0.030 (-0.053, -0.006)	0.015	0.006 (-0.016, 0.027)	0.617
Regular exercise	-0.019 (-0.042, 0.004)	0.114	-0.007 (-0.029, 0.015)	0.547
Sleeping well	-0.024 (-0.044, -0.004)	0.017	-0.020 (-0.038, -0.002)	0.034
Alcohol drinking	-0.029 (-0.049, -0.009)	0.005	-0.005 (-0.023, 0.013)	0.585

Values are presented as mean (95% confidence interval).

1 A company conducted a survey using questionnaires to gather information about the percentage of employees who were satisfied with various lifestyle factors, those who had mental health-related absenteeism, and turnover rates among the employees; Using these data, we investigated the associations between employee lifestyles and turnover rates through linear regression models; In each model, regression coefficients and 95% confidence intervals were computed for various lifestyle indicators; These estimates indicate how the employee turnover rate varied with a 1 percentage point increase in the proportion of employees who maintained a healthy weight, smoked, exercised regularly, slept well, and consumed alcohol; The adjusted model encompassed all lifestyle factors and confounders, including listed company (yes/no), type of industry (retail or service/wholesale or others), and length of service (continuous variable).

**Table 2.** Associations of employee lifestyle with mental health-related absenteeism among 1,748 Japanese companies^1^

Variables (1%)	Unadjusted model	p-value	Adjusted model	p-value
Maintenance of appropriate weight	0.003 (-0.004, 0.010)	0.347	-0.002 (-0.009, 0.005)	0.504
Smoking	-0.015 (-0.020, -0.011)	<0.001	-0.013 (-0.017, -0.008)	<0.001
Regular exercise	-0.008 (-0.012, -0.003)	<0.001	-0.005 (-0.010, -0.001)	0.021
Sleeping well	-0.006 (-0.010, -0.002)	0.002	-0.005 (-0.009, -0.001)	0.016
Alcohol drinking	-0.008 (-0.012, -0.004)	<0.001	-0.004 (-0.007, 0.000)	0.068

Values are presented as mean (95% confidence interval).

1 A company conducted a survey using questionnaires to gather information about the percentage of employees who were satisfied with various lifestyle factors, those who had mental health-related absenteeism, and turnover rates among the employees; Using these data, we investigated the associations between employee lifestyles and turnover rates through linear regression models; In each model, regression coefficients and 95% confidence intervals were computed for various lifestyle indicators; These estimates indicate how the employee turnover rate varied with a 1 percentage point increase in the proportion of employees who maintained a healthy weight, smoked, exercised regularly, slept well, and consumed alcohol; The adjusted model encompassed all lifestyle factors and confounders, including listed company (yes/no), type of industry (retail or service/wholesale or others), and length of service (continuous variable).
